# Nonnegative matrix factorization‐based bioinformatics analysis reveals that TPX2 and SELENBP1 are two predictors of the inner sub‐consensuses of lung adenocarcinoma

**DOI:** 10.1002/cam4.4386

**Published:** 2021-11-03

**Authors:** Haiwei Wang, Xinrui Wang, Liangpu Xu, Hua Cao, Ji Zhang

**Affiliations:** ^1^ Fujian Key Laboratory for Prenatal Diagnosis and Birth Defect Fujian Maternity and Child Health Hospital Affiliated Hospital of Fujian Medical University Fuzhou Fujian China; ^2^ Key Laboratory of Technical Evaluation of Fertility Regulation for Non‐human Primate National Health and Family Planning Commission Fuzhou Fujian China; ^3^ State Key Laboratory for Medical Genomics Shanghai Institute of Hematology Rui‐Jin Hospital Affiliated to School of Medicine Shanghai Jiao Tong University Shanghai China

**Keywords:** nonnegative matrix factorization, SELENBP1, sub‐consensus of lung adenocarcinoma, TPX2

## Abstract

**Background:**

Lung adenocarcinoma (LUAD) is a heterogeneous disease. However the inner sub‐groups of LUAD have not been fully studied. Markers predicted the sub‐groups and prognosis of LUAD are badly needed.

**Aims:**

To identify biomarkers associated with the sub‐groups and prognosis of LUAD.

**Materials and Methods:**

Using nonnegative matrix factorization (NMF) clustering, LUAD patients from The Cancer Genome Atlas (TCGA), Gene Expression Omnibus (GEO) datasets and LUAD cell lines from Genomics of Drug Sensitivity in Cancer (GDSC) dataset were divided into different sub‐consensuses based on the gene expression profiling. The overall survival of LUAD patients in each sub‐consensus was determined by Kaplan‐Meier survival analysis. The common genes which were differentially expressed in each sub‐consensus of LUAD patients and LUAD cell lines were identified using TBtools. The predictive accuracy of TPX2 and SELENBP1 for theinner sub‐consensuses of LUAD was determined by Receiver operator characteristic (ROC) analysis. The Kaplan‐Meier survival analysis was also used to test the prognostic significance of TPX2 and SELENBP1 in LUAD patients.

**Results:**

Using nonnegative matrix factorization clustering, LUAD patients in The Cancer Genome Atlas (TCGA), GSE30219, GSE42127, GSE50081, GSE68465, and GSE72094 datasets were divided into three sub‐consensuses. Sub‐consensus3 LUAD patients were with low overall survival and were with high TP53 mutations. Similarly, LUAD cell lines were also divided into three sub‐consensuses by NMF method, and sub‐consensus2 cell lines were resistant to EGFR inhibitors. Identification of the common genes which were differentially expressed in different sub‐consensuses of LUAD patients and LUAD cell lines revealed that TPX2 was highly expressed in sub‐consensus3 LUAD patients and sub‐consensus2 LUAD cell lines. On the contrary, SELENBP1 was highly expressed in sub‐consensus1 LUAD patients and sub‐consensus1 LUAD cell lines. The expression levels of TPX2 and SELENBP1 could distinguish sub‐consensus3 LUAD patients or sub‐consensus2 LUAD cell lines from other sub‐consensuses of LUAD patients or cell lines. Moreover, compared with normal lung tissues, TPX2 was highly expressed, while, SELENBP1 was lowly expressed in LUAD tissues. Furthermore, the higher expression levels of TPX2 were associated with the lower relapse‐free survival and the lower overall survival of LUAD patients. While, the higher expression levels of SELENBP1 were associated with the higher relapse‐free survival and higher overall survival. At last, we showed that TP53 mutant LUAD patients were with higher TPX2 and lower SELENBP1 expressions.

**Discussion:**

Both iCluster and NMF method are proved to be robust LUAD classification systems. However, the LUAD patients in different iclusters had no significant clinical overall survival, while, sub‐consensus3 LUAD patients from NMF classification were with lower overall survival than other sub‐consensuses.

**Conclusions:**

By integrated analysis of 1765 LUAD patients and 64 LUAD cell lines, we showed that NMF was a robust inner sub‐consensuses classification method of LUAD. TPX2 and SELENBP1 were differentially expressed in different LUAD sub‐ consensuses, and predicted the inner sub‐consensuses of LUAD with high accuracy. TPX2 was an unfavorable prognostic biomarker of LUAD which was up‐regulated in LUAD tissues and associated with the low overall survival of LUAD. SELENBP1 was a favorable prognostic biomarker of LUAD which was down‐regulated in LUAD tissues and associated with the prolonged overall survival of LUAD.

## Graphical abstract

NMF is a robust method for the inner sub‐consensuses classification of LUAD. TPX2 and SELENBP1 are two predictors of the inner sub‐consensuses of LUAD.

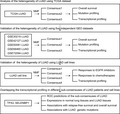

## 1 BACKGROUND

Lung adenocarcinoma (LUAD) is one of common and lethal type of non‐small cell lung cancer (NSCLC).^1^, ^2^ The incidence and mortality of LUAD are increasing every year.^3^, ^4^ Genetic alterations of LUAD are extensively studied. Somatically mutant tumor suppressor gene TP53 and activated oncogenes EGFR and KRAS are commonly detected in LUAD.^5^ Although molecular‐targeted therapies, like EGFR inhibition therapy^6^, ^7^ and checkpoint blockade immune therapy^8^, ^9^ have achieved some improvements in clinical outcomes, the 5‐year survival rate of LUAD remains very low. LUAD is a heterogeneous disease. Previously, based on the genetic alterations,^10^, ^11^ the mRNA,^12^, ^13^, ^14^ microRNA^15^, ^16^, ^17^ or long non‐coding RNA expression signature,^18^, ^19^, ^20^ and the immune cells infiltration signature,^21^, ^22^ LUAD could be further divided into different sub‐groups. In the year of 2014, the Cancer Genome Atlas (TCGA) research groups divided LUAD into six clusters using iCluster analysis by integrating the gene expression, DNA methylation, and genetic alterations of LUAD.^5^ Each cluster of LUAD showed different molecular features. For example, TP53 mutations were enriched in clusters 1–3 and SETD2 mutations were enriched in cluster 4. This study provided deep understanding of the molecular heterogeneity of LUAD. However, the clinical overall survival of those six clusters was not significantly different. So, new classification methods are needed to further reveal the inner sub‐consensuses of LUAD. And more prognostic makers are needed to predict the therapeutic responses and clinical outcomes of LUAD.

Nonnegative matrix factorization (NMF) is an un‐supervised sub‐consensus clustering system.^23^ By calculating the approximation of the factors, NMF could reveal the basic patterns of multidimensional data.^24^, ^25^ NMF has been used successfully in many fields, including cancers. Colon cancer patients could be divided into goblet‐like, enterocyte, stem‐like, inflammatory, and transit‐amplifying five sub‐consensuses based on NMF classification.^26^ And this sub‐consensus classification system could predict the clinical outcomes and therapeutic responses of colon cancer patients. We also used NMF method to identify the sub‐consensus of colon cancer cell lines and found a sub‐consensus of colon cancer cells was sensitive to BRAF inhibitors and PI3K‐mTOR inhibitors.^27^ NMF was also used for liver cancer classification^28^, ^29^, ^30^ and pancreatic cancer classification.^31^, ^32^ Lung squamous cell carcinoma (LUSC) is another type of NSCLC.^33^ Using NMF method, LUSC could be divided into LUSC‐A and LUSC‐B two sub‐consensuses with different biological characteristics and clinical outcomes.^34^ All those results highlighted that NMF was a robust cancer classification system. However, the robustness of NMF classification system in LUAD was not analyzed.

To the best of our knowledge, this is the first integrated bioinformatics study of large cohorts of LUAD patients and LUAD cell lines to reveal the inner heterogeneity of LUAD by NMF method. Our results provide deep understanding of the inner heterogeneity of LUAD. Our data also suggests the potential prognostic biomarkers and therapeutic targets of LUAD.

## 2 MATERIALS AND METHODS

### 2.1 Data collection and processing

TCGA LUAD gene expression dataset, DNA mutation dataset, and LUAD clinical dataset were downloaded from the TCGA hub (https://tcga.xenahubs.net).^5^

The gene expression matrix of LUAD patients along with the clinical survival information was downloaded from the Gene Expression Omnibus (GEO) website (www.ncbi.nlm.nih.gov/geo), including GSE30219,^35^ GSE42127,^36^ GSE50081,^37^ GSE68465,^38^ and GSE72094 ^39^ datasets. The gene expression series matrix of normal lung tissues and LUAD tissues was downloaded from GSE7670,^40^ GSE10072,^41^ GSE18842,^42^ GSE27262,^43^ and GSE32863 ^44^ datasets. Raw CEL data and clinical data of LUAD patients in MSKCC dataset 1 and MSKCC dataset 2 were available athttp://cbio.mskcc.org/Public/lung_array_data/.^45^ All the GEO datasets were annotated using R software (version 3.5.0). The expression values were averaged by “plyr” package (version 1.8.5).

Gene expression matrix and drug sensitivity of LUAD cell lines were downloaded from Genomics of Drug Sensitivity in Cancer (GDSC) project (https://www.cancerrxgene.org/).^46^

### 2.2 NMF classification of LUAD patients and LUAD cell lines

LUAD patients and LUAD cell lines were divided into two sub‐consensuses, three sub‐consensuses, or four sub‐consensuses by “NMF” package in R software based on the globe gene expression levels (version 0.23.0l). The number of sub‐consensuses was determined by the number of ranks.

### 2.3 Survival analysis

The overall survival of LUAD patients in each sub‐consensus was determined by the Kaplan–Meier survival analysis which was performed using “survival” package (version 3.1‐8) in R statistics software. The prognostic values of TPX2 and SELENBP1 on the relapse‐free survival or overall survival were also determined using “survival” package. *P* values were calculated by the log‐rank test.

### 2.4 Heatmap presentation

The heatmaps were generated using “pheatmap” package (version 1.0.12) in R statistics software. The “average” method determined the clustering scale and “correlation” method determined the clustering distance.

### 2.5 Venn diagram

The Venn diagram was generated using Wonderful Venn in TBtools software (version x32_1_064).^47^

### 2.6 ROC analysis

The ROC curves were plotted by “pROC” package (version 1.16.2) in R statistics software. The area under the ROC curve (AUC) was also calculated by “pROC” package.

### 2.7 Biological process enrichment analysis

The enriched biological process was identified using The Database for Annotation, Visualization, and Integrated Discovery (DAVID) website (version 6.8; https://david.ncifcrf.gov).^48^, ^49^ The enriched biological processes with *p* values < 0.05 were considered to be statistically significant.

### 2.8 Statistical analysis

The box plots were generated from GraphPad Prism software (version 5.0). Statistical analysis was performed using the two‐tailed paired Student's *t*‐test. *P* value <0.05 was chosen to be significantly different.

## 3 RESULTS

### 3.1 Identification of the molecular sub‐consensuses of LUAD by NMF method using TCGA dataset

**FIGURE 1:**
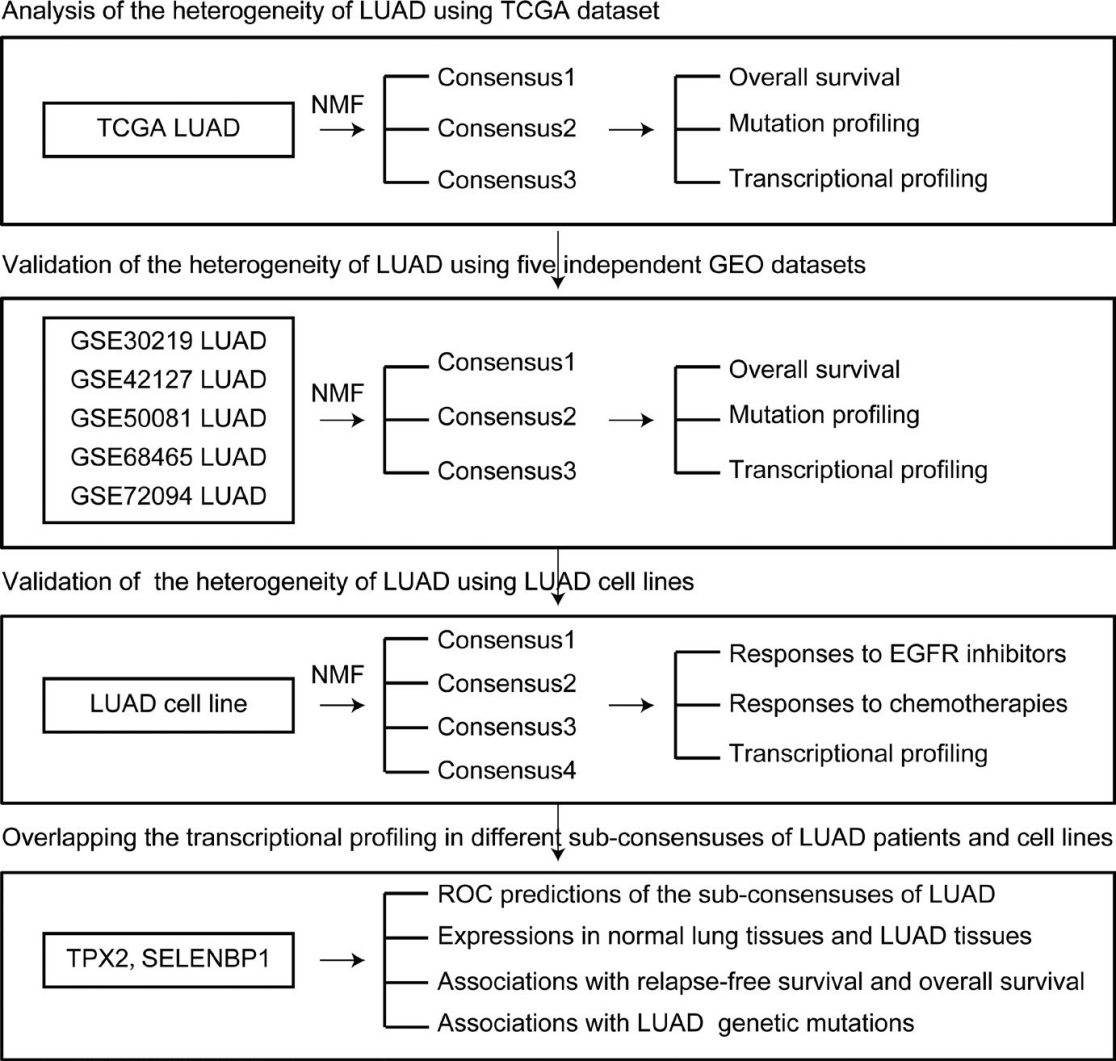
A working process to study the molecular sub‐consensuses of LUAD based on NMF classification

**FIGURE 2:**
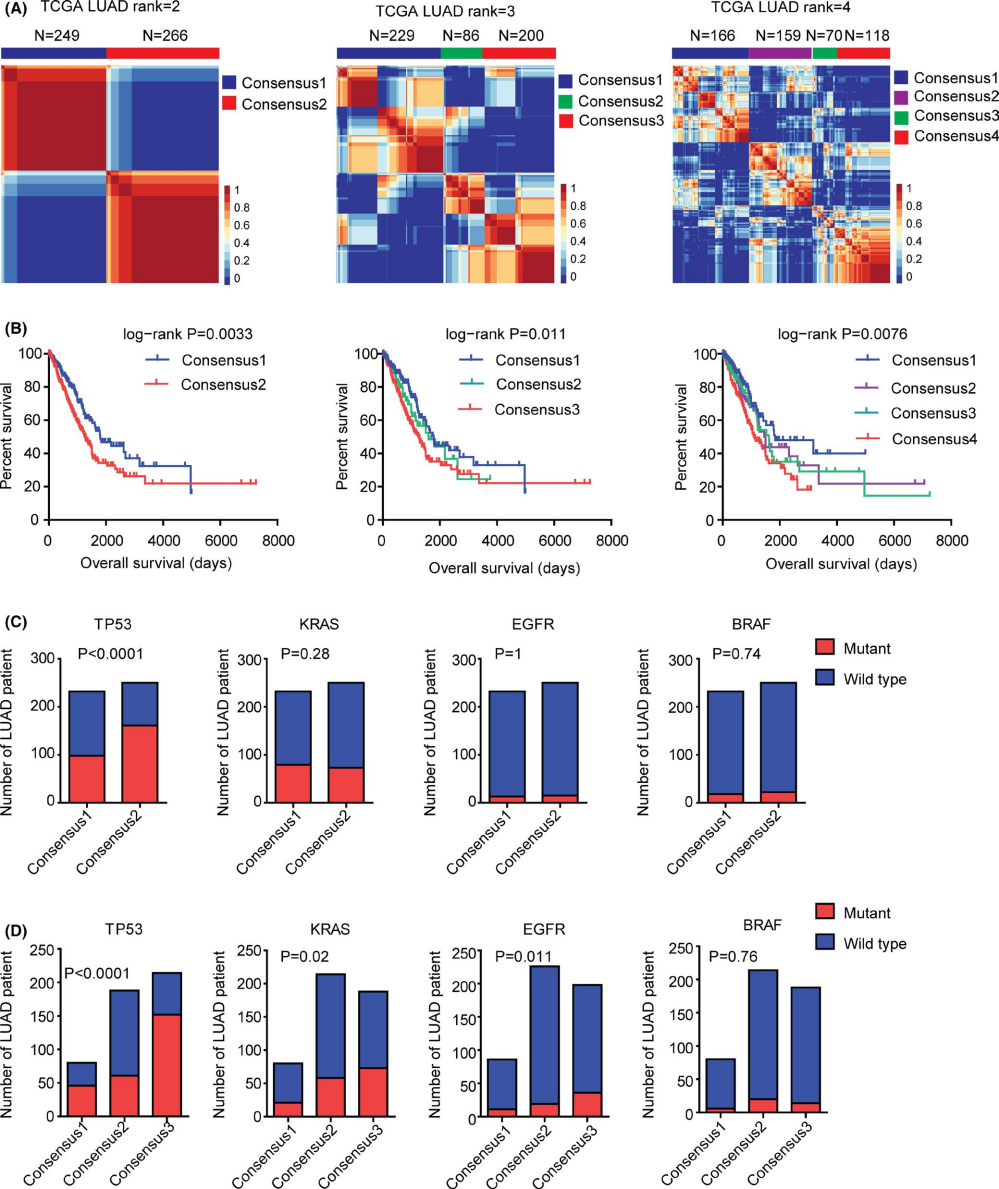
Identification of the molecular sub‐consensuses of LUAD by NMF method using TCGA dataset. (A) Primary LUAD patients from TCGA dataset were divided into two, three, or four sub‐consensuses based on the gene transcriptional profiling using NMF method. Consensus maps showed the correlation profiling of LUAD derived from two sub‐consensuses, three sub‐consensuses, or four sub‐consensuses. (B) The Kaplan–Meier survival analysis was used to determine the overall survival of LUAD patients in each sub‐consensus derived from two, three, or four sub‐consensuses classification. The overall survival *p* values were calculated by the log‐rank test. (C) Contingency graphs showed the number of LUAD patients with TP53, KRAS, EGFR, or BRAF mutations in each sub‐consensus derived from the two sub‐consensuses classification. *p* values were determined using the Chi‐squared test. (D) Contingency graphs showed the number of LUAD patients with TP53, KRAS, EGFR, or BRAF mutations in each sub‐consensus derived from the three sub‐consensuses classification

We designed a working process to study the molecular sub‐consensuses of LUAD based on NMF classification (Figure 1). First, 515 LUAD patients were collected from TCGA dataset.^5^ Based on the gene transcriptional profiling, those patients were divided into two sub‐consensuses using NMF method. In total, 249 LUAD patients were in sub‐consensus 1 and 266 LAUD patients were in sub‐consensus 2. Consensus map showed the high correlation of LUAD patients in each sub‐consensus (Figure 2A). Moreover, we found significantly different overall survival in sub‐consensus 1 and sub‐consensus 2 LUAD patients. LUAD patients in sub‐consensus 2 had lower overall survival (Figure 2B). Furthermore, LUAD patients in sub‐consensus 2 were with higher TP53 mutations (Figure 2C). However, the KRAS, EGFR, or BRAF mutations in sub‐consensus 1 and sub‐consensus 2 were not significantly different (Figure 2C).

One advantage of NMF classification is that the number of sub‐consensuses can be easily determined by the number of ranks.^34^ We further divided the 515 LUAD patients into three or four sub‐consensuses using NMF method (Figure 2A). Overall survival was significantly different in different sub‐consensuses of LUAD patients (Figure 2B). LUAD patients in sub‐consensus 3 had the lowest overall survival. TP53, KRAS, and EGFR mutations, but not BRAF mutations, were significantly higher in sub‐consensus 3 of LUAD patients (Figure 2D). Also, in the four sub‐consensuses classification, compared with other sub‐consensuses, LUAD patients in sub‐consensus 4 had the lowest overall survival (Figure 2B). Those results suggested that, using NMF method, LUAD patients from TCGA dataset could be divided into different sub‐consensuses with different molecular characteristics and clinical overall survival.

### 3.2 Validation of the sub‐consensuses classification of LUAD using five independent GEO datasets

Using the expression datasets deposited in GEO website, we further validated the sub‐consensuses NMF classification of LUAD. Collectively, 85 LUAD patients from GSE30219,^35^ 133 LUAD patients from GSE42127,^36^ 127 LUAD patients from GSE50081,^37^ 462 LUAD patients from GSE68465,^38^ and 442 LUAD patients from GSE72094 datasets^39^ were used for further studies. Similarly, using NMF classification, LUAD patients in each GEO dataset were divided into two sub‐consensuses (Figure S1A). Then, the overall survival of each sub‐consensus of LUAD patients was determined. LUAD patient in sub‐consensus 2 had more unfavorable prognosis than LUAD patients in sub‐consensus 1 in GSE30219, GSE50081, GSE68465, and GSE72094 datasets (Figure S1B).

**FIGURE 3:**
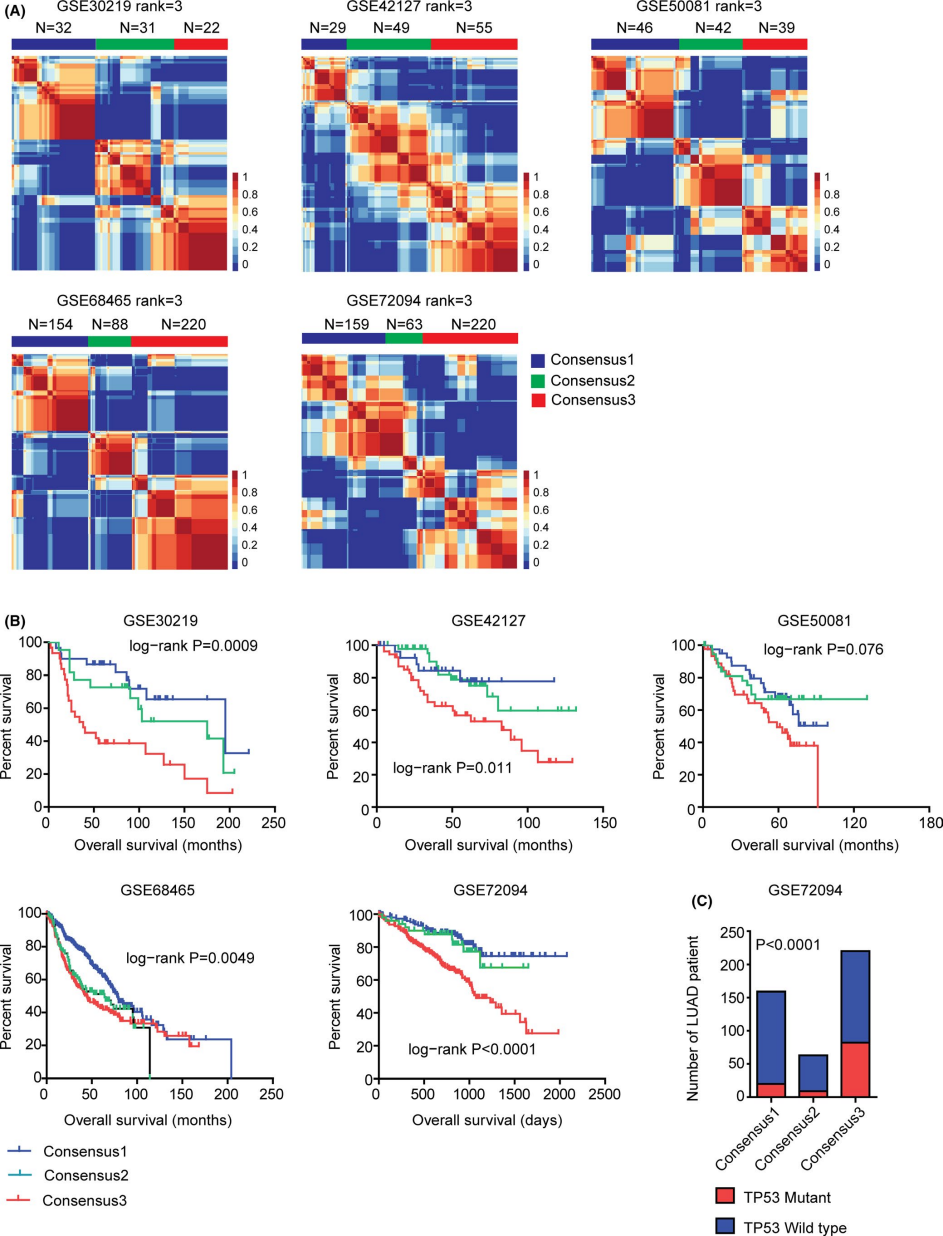
Validation of the sub‐consensuses classification of LUAD using five independent GEO datasets. (A) Primary LUAD patients from GSE30219, GSE42127, GSE50081, GSE68465, and GSE72094 datasets were divided into three sub‐consensuses based on the gene expression profiling. (B) The Kaplan–Meier survival analysis was used to determine the different overall survival of sub‐consensus 1, sub‐consensus 2, and sub‐consensus 3 LUAD patients derived from GSE30219, GSE42127, GSE50081, GSE68465, and GSE72094 datasets. The overall survival *p* values were determined by the log‐rank test. (C) Contingency graphs showed the number of LUAD patients with TP53 mutations in each sub‐consensus derived from the three sub‐consensuses classification in GSE72094 dataset

Using same strategy, LUAD patients derived from GSE30219, GSE42127, GSE50081, GSE68465, and GSE72094 datasets were divided into three sub‐consensuses, as illustrated in the consensus maps (Figure 3A). Similar to the results derived from TCGA dataset, the Kaplan–Meier survival analysis showed that LUAD patient in sub‐consensus 3 had the worst overall survival than LUAD patients in other sub‐consensuses in GSE30219, GSE42127, GSE50081, GSE68465, and GSE72094 datasets (Figure 3B). Furthermore, LUAD patients in sub‐consensus 3 were with higher TP53 mutations in GSE72094 dataset (Figure 3C).

So, by integrated analysis of total 1765 LUAD patients from TCGA and GEO datasets, we concluded that three sub‐consensuses of NMF method was a robust classification method of LUAD.

### 3.3 Validation of the sub‐consensuses classification of LUAD using LUAD cell lines

Primary LUAD tissues include LUAD tumor cells, tumor stroma cells, and infiltrated immune cells. The tissue complexity may influence the classification of LUAD. On the contrary, LUAD cell lines are relatively homogenous, and the expression profiling of cell lines may represent the intrinsic sub‐consensus of LUAD. So, we used LUAD cell lines to validate the three sub‐consensuses classification of primary LUAD patients.

**FIGURE 4:**
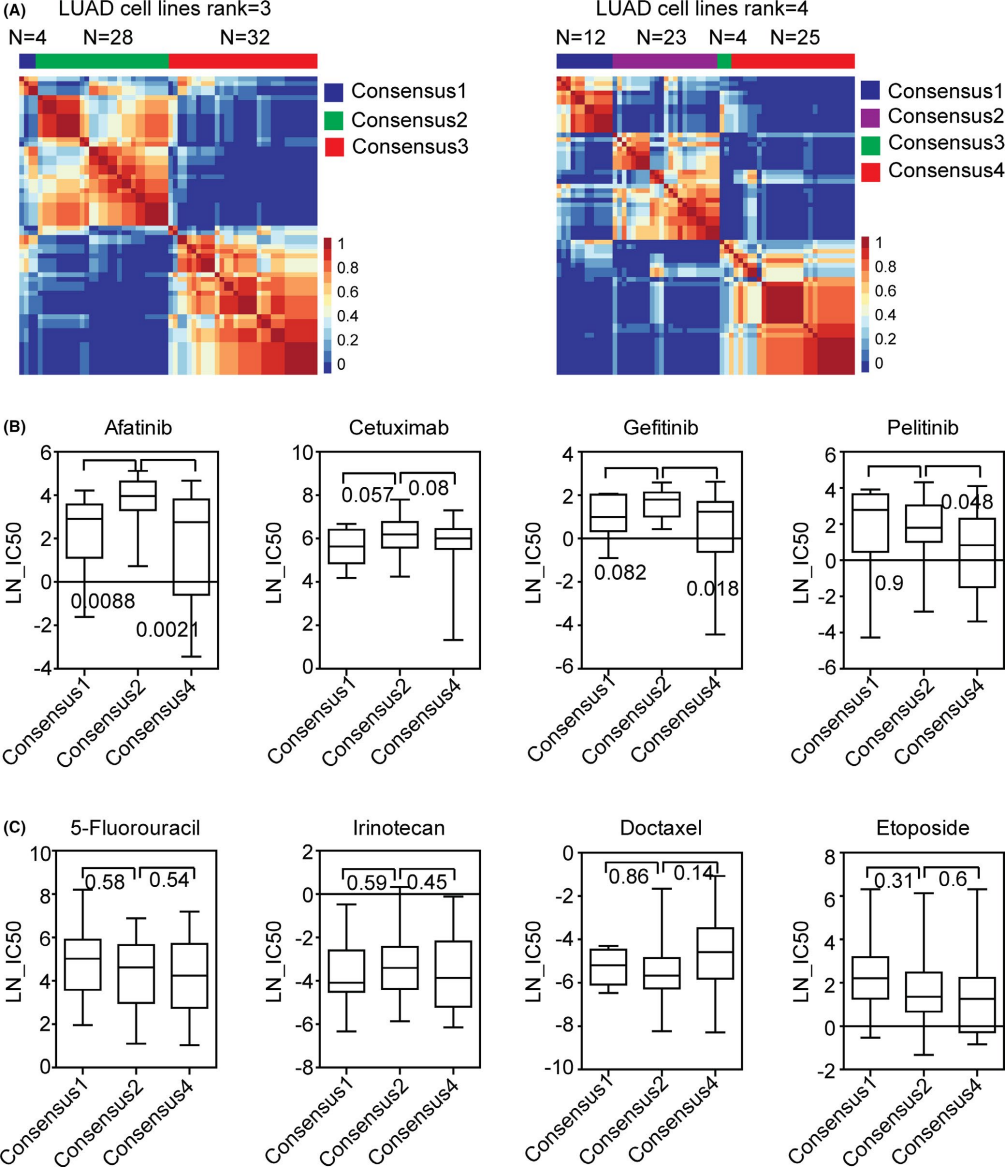
Validation of the sub‐consensuses classification of LUAD using LUAD cell lines. (A) LUAD cell lines were divided into three or four sub‐consensuses based on the gene expression profiling using NMF. Consensus maps showed the correlation profiling of LUAD cell lines from three sub‐consensuses or four sub‐consensuses. (B) Box plots showed the LN‐IC50 of EGFR inhibitors afatinib, cetuximab, gefitinib, and pelitinib in each LUAD sub‐consensus of cell lines derived from the four sub‐consensuses classification. *P* values were generated by two‐tailed paired Student's *t*‐test. (C) Box plots showed the LN‐IC50 of chemotherapeutic drugs 5‐fluorouracil, irinotecan, docetaxel, and etoposide in each LUAD sub‐consensus of cell lines derived from the four sub‐consensuses classification

Gene expression data and drug responses of 64 LUAD cell lines were downloaded from Genomics of Drug Sensitivity in Cancer (GDSC) project.^46^ First, the 64 LUAD cell lines were divided into two (Figure S2A), three, or four sub‐consensuses based on the gene expression profiling using NMF method (Figure 4A). Then, we tested the cell responses to EGFR inhibitors and chemotherapeutic drugs in different LUAD sub‐consensuses. When the LUAD cell lines were classified into two sub‐consensuses, cells in sub‐consensus 1 were more sensitive to EGFR inhibitors afatinib and gefitinib (Figure S2B). However, there was no significant difference in the cetuximab and pelitinib sensitivity between sub‐consensus 1 and sub‐consensus 2 LUAD cells (Figure S2B).

We then divided the LUAD cell lines into three or four sub‐consensuses. However, the number of cell lines of sub‐consensus 1 in the three sub‐consensuses classification and sub‐consensus 3 in the four sub‐consensuses classification was very low (Figure 4A). Therefore, only the sub‐consensus 1, 2, and 4 in the four sub‐consensuses classification were further studied. LUAD cells in sub‐consensus 2 were more resistant to EGFR inhibitors afatinib, cetuximab, gefitinib, and pelitinib treatment compared with LUAD cells in sub‐consensus 1 and sub‐consensus 4 (Figure 4B). However, there was no significant difference in the 5‐fluorouracil, irinotecan, docetaxel, and etoposide sensitivity in different LUAD sub‐consensuses (Figure 4C).

### 3.4 Transcriptional characteristics of the sub‐consensus 3 LUAD

**FIGURE 5:**
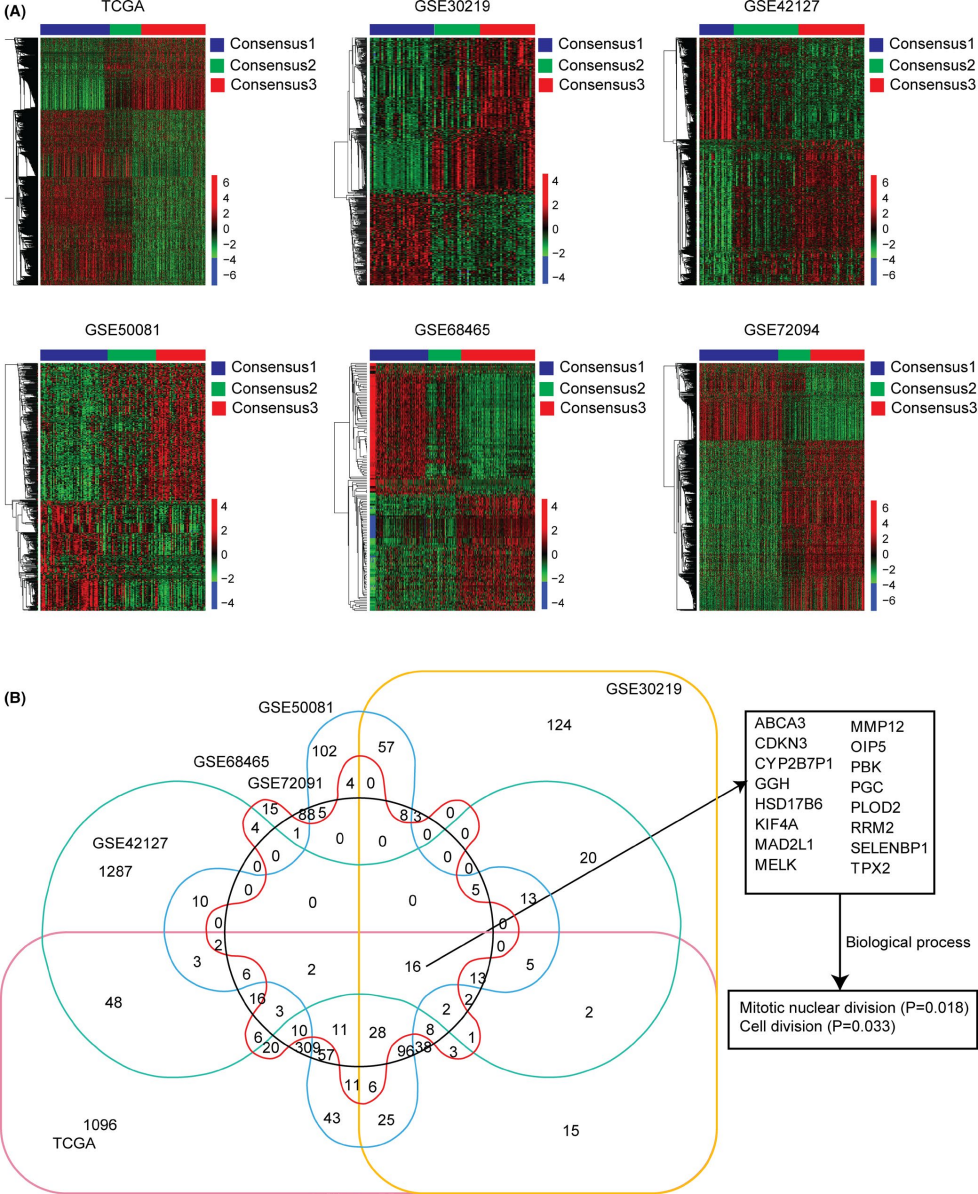
Transcriptional characteristics of the sub‐consensus 3 LUAD. (A) Clustering heatmaps showed the differentially expressed genes between sub‐consensus 1 and sub‐consensus 3 LUAD patients in TCGA, GSE30219, GSE42127, GSE50081, GSE68465, and GSE72094 datasets. Upregulated (red) and downregulated (blue) genes in sub‐consensus 3 LUAD patients are shown. (B) Venn diagram showed the common genes which were differentially expressed between sub‐consensus 1 and sub‐consensus 3 LUAD patients in TCGA, GSE30219, GSE42127, GSE50081, GSE68465, and GSE72094 datasets. The enriched biological processes of the 16 commonly and differentially expressed genes were determined

In all the TCGA, GSE30219, GSE42127, GSE50081, GSE68465, and GSE72094 datasets, the sub‐consensus 3 LUAD patients had unfavorable prognosis, while, the sub‐consensus 1 LUAD patients had favorable prognosis. Based on the absolute fold changes >2 and *p* values <0.001 criterion, the differentially expressed genes between sub‐consensus 1 and sub‐consensus 3 were determined. As demonstrated in the clustering heatmaps, 1903 genes were differentially expressed in TCGA dataset, 490 genes were differentially expressed in GSE30219 dataset, 1466 genes were differentially expressed in GSE42127 dataset, 528 genes were differentially expressed in GSE50081 dataset, 153 genes were differentially expressed in GSE68465 dataset, and 727 genes were differentially expressed in GSE72094 dataset, respectively (Figure 5A).

Next, using overlapping analysis, we tried to identify the common genes which were differentially expressed in sub‐consensus 3 LUAD patients. As shown in the Venn diagram, 16 genes were differentially expressed between sub‐consensus 1 and sub‐consensus 3 LUAD patients in TCGA, GSE30219, GSE42127, GSE50081, GSE68465, and GSE72094 datasets (Figure 5B). The expression levels of those 16 genes were further demonstrated in heatmaps (Figure S3). In TCGA, GSE30219, GSE42127, GSE50081, GSE68465, and GSE72094 datasets, MMP12, PLOD2, GGH, OIP5, PBK, RRM2, MELK, KIF4A, TPX2, CDKN3, and MAD2L1 were all upregulated in sub‐consensus 3 LUAD patients, while, HSD17B6, CYP2B7P1, SELENBP1, ABCA3, and PGC were all downregulated in sub‐consensus 3 LUAD patients. Biological process enrichment analysis showed that those 16 genes were associated with mitotic nuclear division and cell division processes (Figure 5B).

### 3.5 TPX2 and SELENBP1 are differentially expressed in different LUAD sub‐consensuses

**FIGURE 6:**
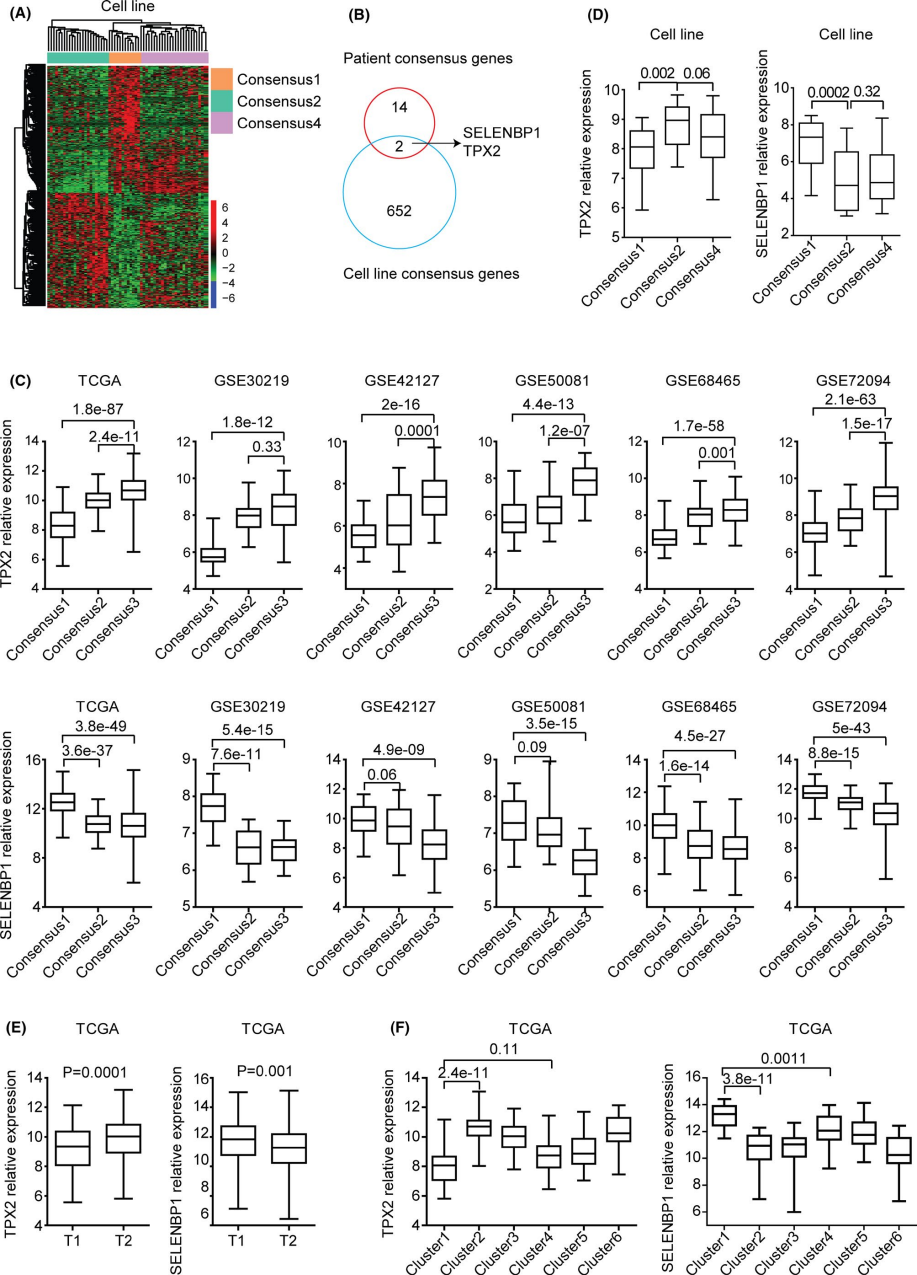
TPX2 and SELENBP1 are differentially expressed in different LUAD sub‐consensuses. (A) Un‐supervised clustering heatmap demonstrated the differentially expressed genes in sub‐consensus 2 LUAD cell lines. (B) Venn diagram depicted the common genes associated with the different sub‐consensuses of LUAD patients and cell lines. (C) Box plots showed the TPX2 and SELENBP2 expression levels in each sub‐consensus of LUAD patients derived from TCGA, GSE30219, GSE42127, GSE50081, GSE68465, and GSE72094 datasets. *p* values were generated using the two‐tailed paired Student's *t*‐test. (D) Box plots showed the TPX2 and SELENBP2 expression levels in each sub‐consensus of LUAD cell lines. (E) Box plots showed the TPX2 and SELENBP2 expression levels in T1 and T2 stage of TCGA LUAD patients. (F) Box plots showed the expression levels of TPX2 and SELENBP2 in each cluster based on the TCGA LUAD iCluster classification

Genes differentially expressed in sub‐consensus 2 LUAD cell lines were further identified. Based on the *p* values <0.001 criterion, 654 genes were differentially expressed in sub‐consensus 2 LUAD cell lines, compared with sub‐consensus 1 LUAD cell lines (Figure 6A). Further overlapping with the genes associated with different sub‐consensuses of LUAD patients, we found that TPX2 and SELENBP1 were two potential predictors of the inner sub‐consensuses of primary LUAD patients and LUAD cell lines (Figure 6B).

TPX2 is a microtubule‐associated gene and is critical to the spindle formation during cell cycle progress.^50^, ^51^ TPX2 was highly expressed in sub‐consensus 3 of LUAD patients in TCGA, GSE30219, GSE42127, GSE50081, GSE68465, and GSE72094 datasets (Figure 6C). On the contrary, SELENBP1 was highly expressed in sub‐consensus 1 of LUAD patients (Figure 6C). Moreover, in the EGFR inhibitors resistant sub‐consensus 2 LUAD cell lines, TPX2 was highly expressed and SELENBP1 was lowly expressed (Figure 6D). Furthermore, compared with T1 stage, the expression levels of TPX2 were relatively higher in T2 stage. While, the expression levels of SELENBP1 were relatively lower in T2 stage of LUAD patients (Figure 6E). Previously, TCGA LUAD research groups used iCluster analysis and divided the TCGA LUAD patients into six clusters.^5^ TPX2 was highly expressed and SELENBP1 was lowly expressed in cluster 1 LUAD patients (Figure 5F).

### 3.6 TPX2 and SELENBP1 are two predictors of the sub‐consensuses of LUAD

**FIGURE 7:**
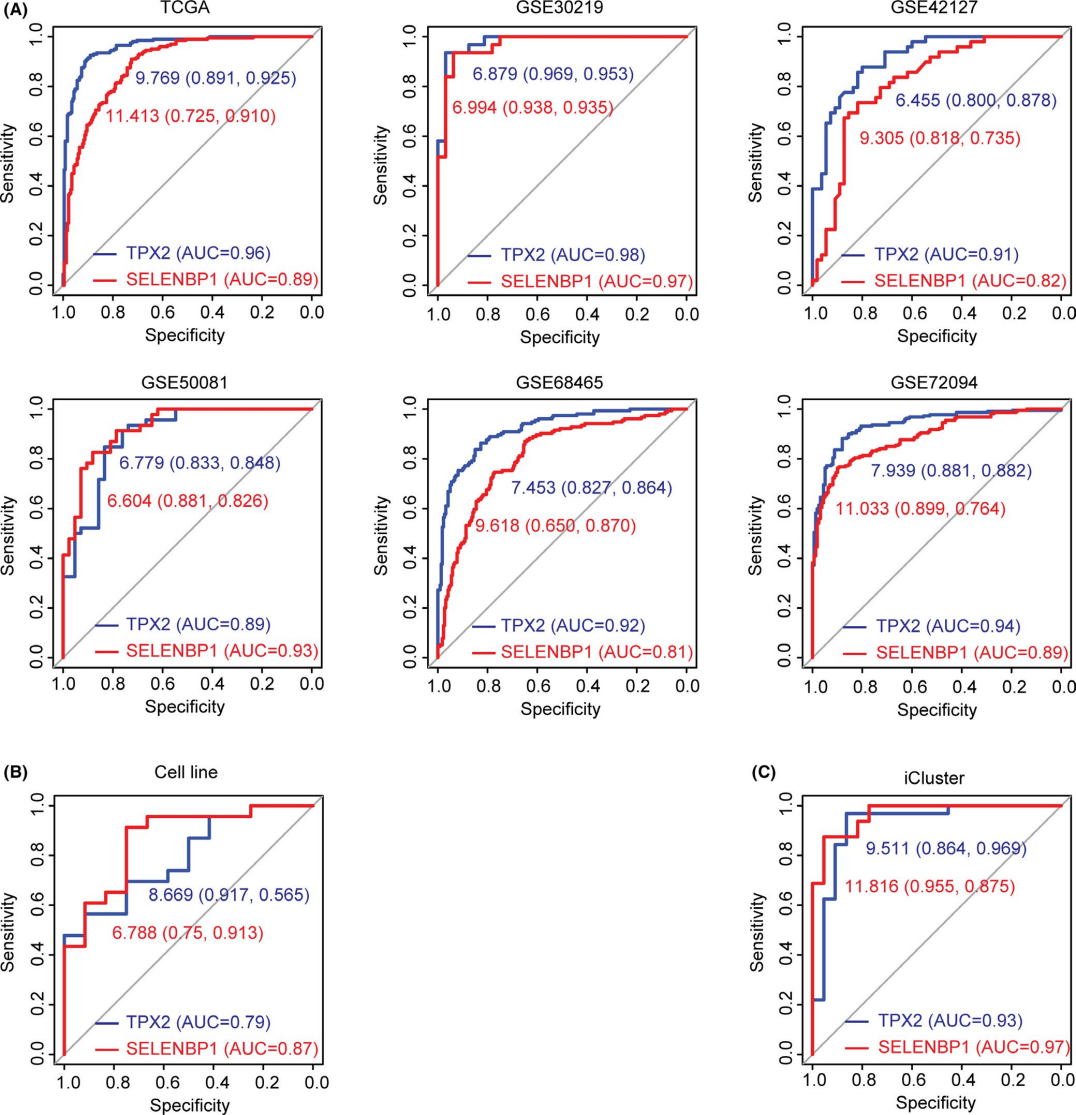
TPX2 and SELENBP1 are two predictors of the sub‐consensuses of LUAD. (A) ROC curves showed the predictive specificity and sensitivity of TPX2 or SELENBP1 to distinguish sub‐consensus 3 from sub‐consensus 1 LUAD patients derived from TCGA, GSE30219, GSE42127, GSE50081, GSE68465, and GSE72094 datasets. (B) ROC curve showed the predictive accuracy of TPX2 or SELENBP1 to distinguish sub‐consensus 2 from sub‐consensus 1 LUAD cell lines. (C) ROC curve showed the predictive values of TPX2 or SELENBP1 in distinguishing cluster 1 from cluster 2 LUAD patients based on the TCGA LUAD iCluster classification. AUC, area under the ROC curve

Furthermore, we attempted to determine the predictive values of TPX2 and SELENBP in distinguishing the sub‐consensuses of LUAD. The ROC analysis in TCGA, GSE30219, GSE42127, GSE50081, GSE68465, and GSE72094 datasets indicated that the expression levels of TPX2 could distinguish sub‐consensus 3 from sub‐consensus 1 LUAD patients with high specificity and sensitivity (Figure 7A). Similar predictive specificity and sensitivity of SELENBP1 in distinguishing sub‐consensus 3 from sub‐consensus 1 LUAD patients were observed in TCGA, GSE30219, GSE42127, GSE50081, GSE68465, and GSE72094 datasets (Figure 7A). Furthermore, the expression levels of TPX2 or SELENBP1 also distinguished sub‐consensus 2 from sub‐consensus 1 of LUAD cell lines with high accuracy (Figure 7B).

Importantly, the expression levels of TPX2 or SELENBP1 had robust predictive values in the iCluster classification. In TCGA LUAD cohort, ROC curves showed the similar specificity and sensitivity of TPX2 or SELENBP1 to distinguish cluster 1 from cluster 2 TCGA LUAD patients (Figure 7C). All those results highlighted the sub‐types predictive values of TPX2 and SELENBP1 in LUAD.

### 3.7 TPX2 is upregulated in LUAD while SELENBP1 is downregulated in LUAD

**FIGURE 8:**
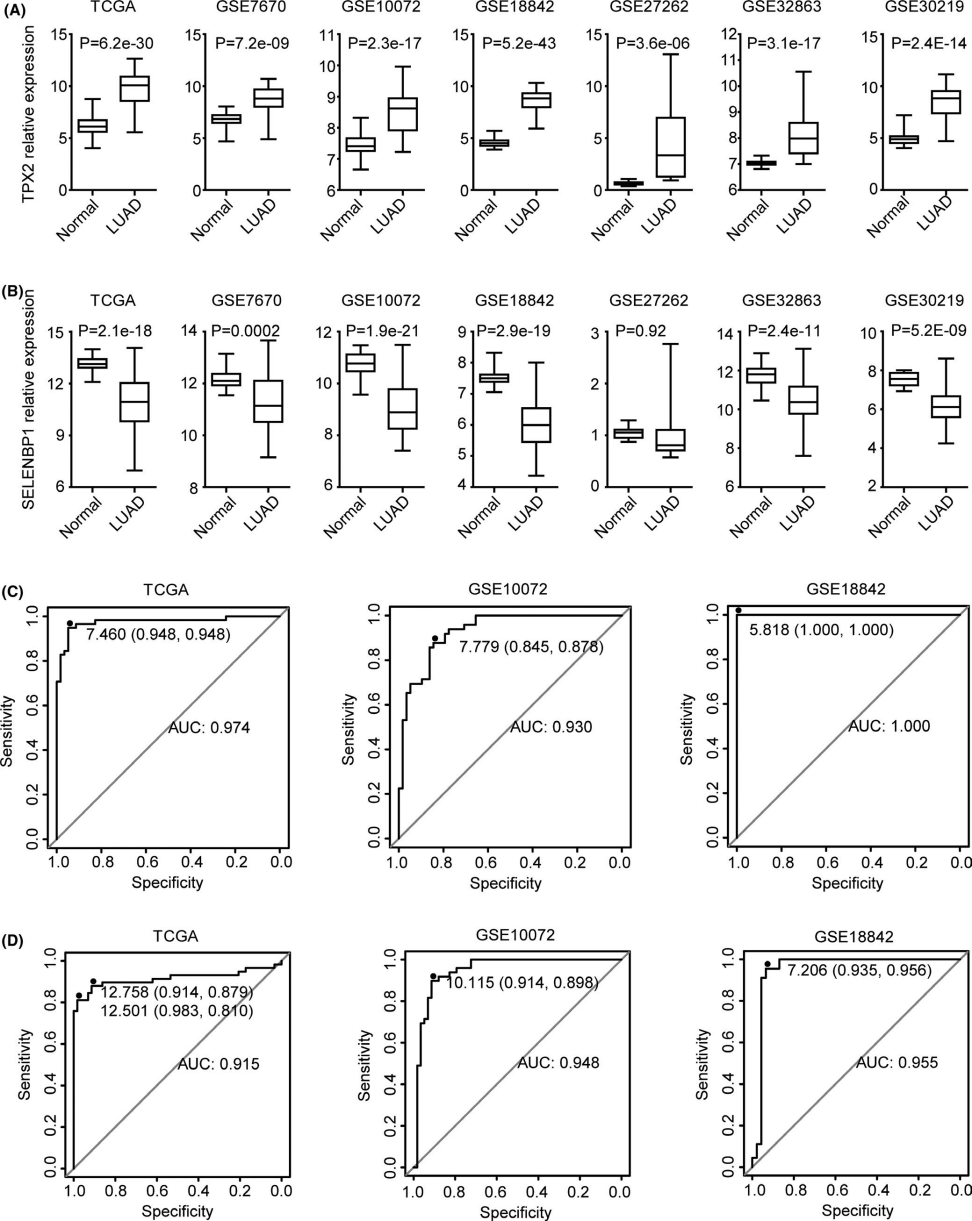
TPX2 is upregulated in LUAD while SELENBP1 is downregulated in LUAD. (A–B) Box plots demonstrated the different expression levels of TPX2 (A) and SELENBP1 (B) in normal lung tissues and LUAD tissues in TCGA, GSE7670, GSE10072, GSE18842, GSE27262, GSE32863, and GSE30219 datasets. *p* values were generated using the two‐tailed paired Student's *t*‐test. (C–D) ROC curves showed the predictive accuracy of TPX2 (C) or SELENBP1 (D) to distinguish LUAD tissues from normal lung tissues in TCGA, GSE10072, and GSE18842 datasets

Next, we analyzed the expression levels of TPX2 and SELENBP1 in normal lung tissues and LUAD tissues. Gene expression profiling of 261 normal lung tissues and 271 LUAD tissues was downloaded from TCGA,^5^ GSE7670,^40^ GSE10072,^41^ GSE18842,^42^ GSE27262,^43^ and GSE32863 ^44^ datasets. In all those six datasets, TPX2 was overexpressed in LUAD tissues, compared with normal lung tissues (Figure 8A). On the contrary, SELENBP1 was significantly downregulated in LUAD tissues in TCGA, GSE7670, GSE10072, GSE18842, and GSE32863 datasets (Figure 8B). Moreover, in GSE30219 dataset, TPX2 was upregulated in LUAD tissues, while SELENBP1 was downregulated in LUAD tissues (Figure 8A,B).

Furthermore, TPX2 and SELENBP1 distinguished LUAD tissues from normal lung tissues with high accuracy. The ROC curve analysis demonstrated significant predictive values of TPX2 in TCGA, GSE10072, and GSE18842 datasets (Figure 8C). Particularly in GSE18842 dataset, the distinguishing of LUAD tissues from normal lung tissues through TPX2 expression was completely accurate (AUC=1). Moreover, high predictive specificity and sensitivity of SELENBP1 in TCGA, GSE10072, and GSE18842 datasets were also observed (Figure 8D).

### 3.8 Higher expression levels of TPX2 are associated with the lower relapse‐free survival and the lower overall survival of LUAD

**FIGURE 9:**
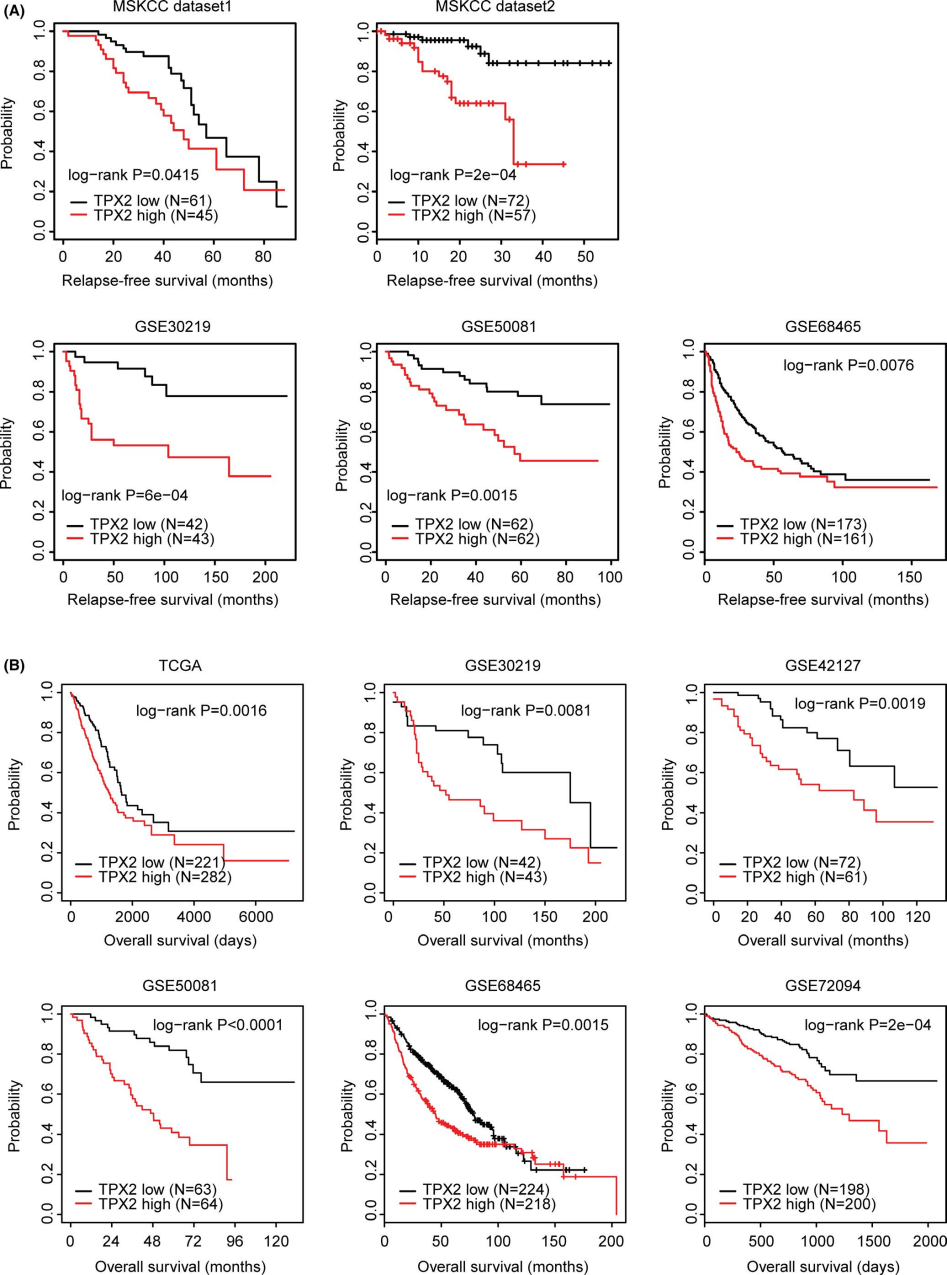
Higher expression levels of TPX2 are associated with the lower relapse‐free survival and the lower overall survival of LUAD. (A) The Kaplan–Meier Plotters demonstrated the associations between TPX2 and the LUAD relapse‐free survival in MSKCC dataset 1, MSKCC dataset 2, GSE30219, GSE50081, and GSE68465 datasets. The *p* values showed the different relapse‐free survival between TPX2 highly expressed LUAD patients (red) and TPX2 lowly expressed LUAD patients (black). (B) The Kaplan–Meier Plotters demonstrated the different overall survival of TPX2 highly expressed LUAD patients (red) and TPX2 lowly expressed LUAD patients (black) in TCGA, GSE30219, GSE42127, GSE50081, GSE68465, and GSE72094 datasets

Then, we determined the prognostic effects of TPX2 in LUAD relapse‐free survival. First, in two MSKCC datasets,^45^ higher expression levels of TPX2 were correlated with lower relapse‐free survival in patients with LUAD (Figure 9A). Furthermore, the unfavorable prognosis of TPX2 in LUAD was validated in GSE30219, GSE50081, and GSE68465 datasets. The Kaplan–Meier Plotters demonstrated that TPX2 highly expressed LUAD patients had lower relapse‐free survival than TPX2 lowly expressed LUAD patients (Figure 9A).

Previously, we showed that TPX2 was highly expressed in sub‐consensus 3 LUAD patients, which had low overall survival in TCGA, GSE30219, GSE42127, GSE50081, GSE68465, and GSE72094 datasets. Consistent with those observations, in all those six datasets, TPX2 highly expressed LUAD patients resulted lower overall survival than TPX2 lowly expressed LUAD patients (Figure 9B), suggesting the importance of TPX2 as a negative marker in the clinical outcome prediction of LUAD.

### 3.9 Lower expression levels of SELENBP1 are associated with the lower relapse‐free survival and the lower overall survival of LUAD

**FIGURE 10:**
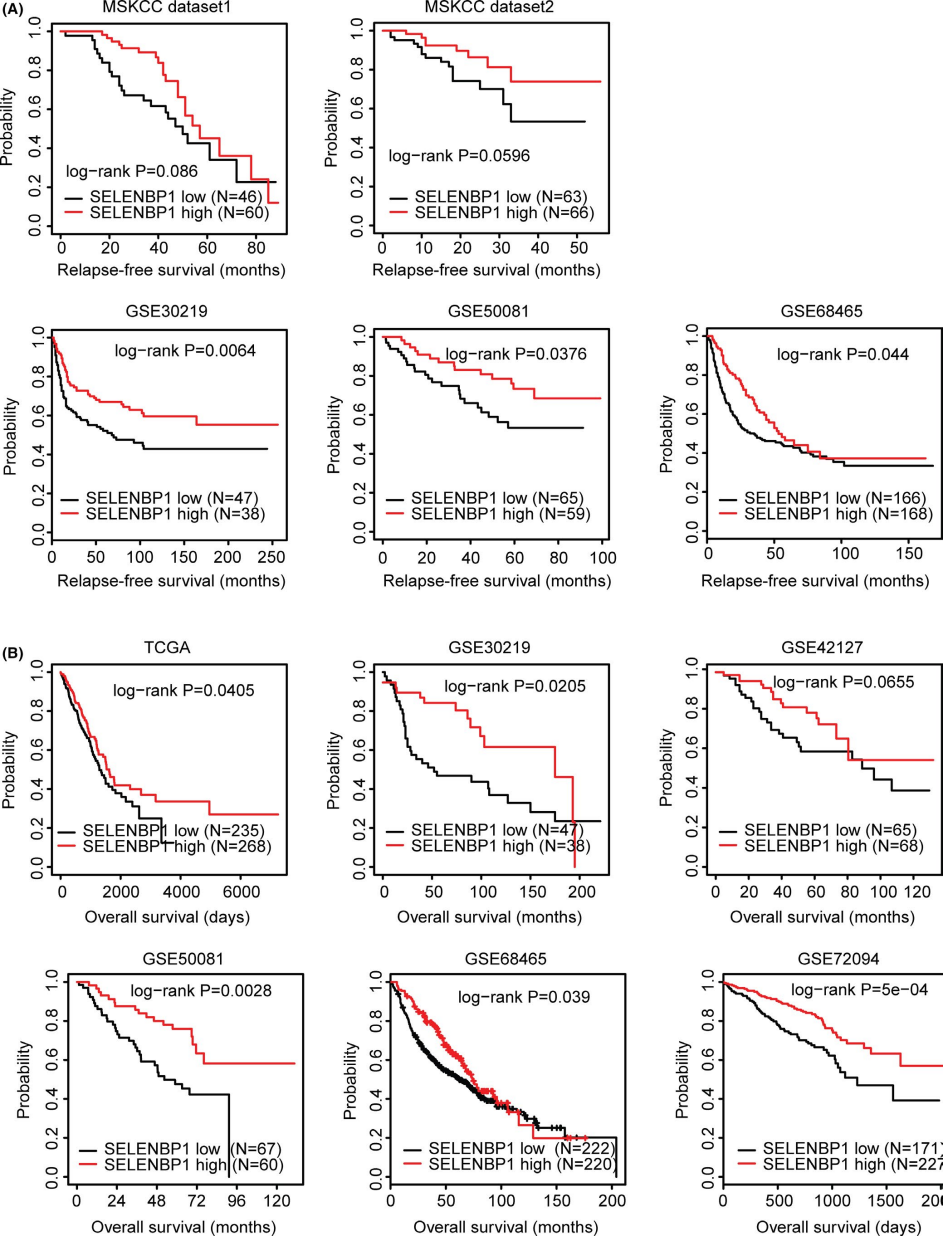
Lower expression levels of SELENBP1 are associated with the lower relapse‐free survival and the lower overall survival of LUAD. (A) The Kaplan–Meier Plotters demonstrated the associations between SELENBP1 and the LUAD relapse‐free survival in MSKCC dataset 1, MSKCC dataset 2, GSE30219, GSE50081, and GSE68465 datasets. The *p* values showed the different relapse‐free survival between SELENBP1 highly expressed LUAD patients (red) and SELENBP1 lowly expressed LUAD patients (black). (B) The Kaplan–Meier Plotters demonstrated the different overall survival of SELENBP1 highly expressed LUAD patients (red) with SELENBP1 lowly expressed LUAD patients (black) in TCGA, GSE30219, GSE42127, GSE50081, GSE68465, and GSE72094 datasets

Unlike TPX2, SELENBP1 may serve as a positive marker in the clinical outcome prediction of LUAD. First, as we previously showed, compared with the normal lung tissues, SELENBP1 was downregulated in LUAD tissues (Figure 8B). Second, LUAD patients with higher expression levels of SELENBP1 were associated with better relapse‐free survival in GSE30219, GSE50081, and GSE68465 datasets (Figure 10A). Less significantly, SELENBP1 highly expressed LUAD patients also had higher relapse‐free survival than SELENBP1 lowly expressed LUAD patients in MSKCC1 and MSKCC2 datasets (Figure 10A).

Third, in TCGA, GSE30219, GSE50081, GSE68465, and GSE72094 datasets, significantly different overall survival between SELENBP1 highly expressed LUAD patients and SELENBP1 lowly expressed LUAD patients was demonstrated (Figure 10B). All those results highlighted the prognostic effects of SELENBP1 in patients with LUAD.

### 3.10 Expression levels of TPX2 and SELENBP1 are correlated with TP53 mutations

**FIGURE 11:**
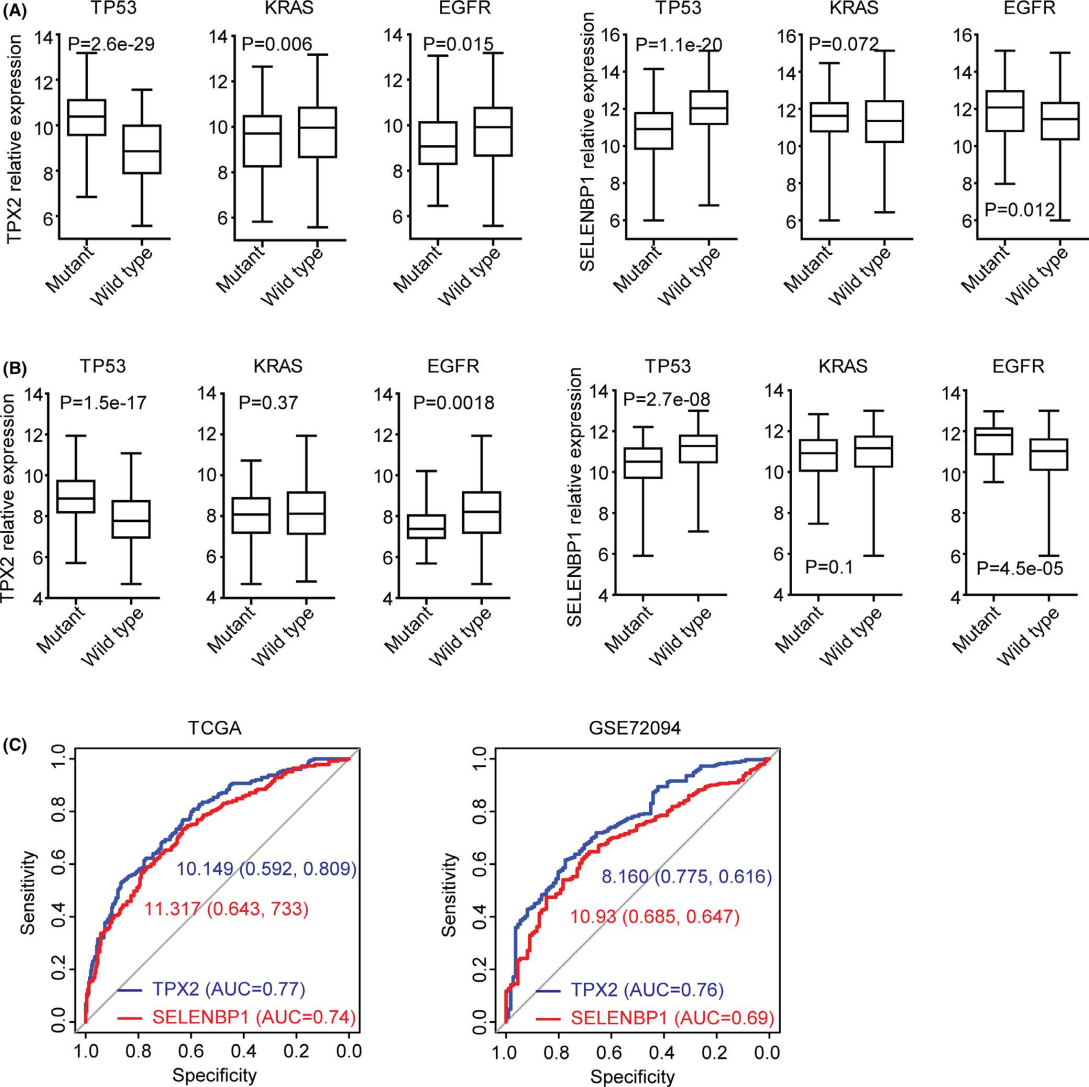
The expression levels of TPX2 and SELENBP1 are correlated with TP53 mutations. (A) The expression levels of TPX2 and SELENBP1 in TP53, KRAS, or EGFR‐mutant LUAD patients and TP53, KRAS, or EGFR wild‐type LUAD patients derived from TCGA dataset. *p* values were generated using the two‐tailed paired Student's *t*‐test. (B) The expression levels of TPX2 and SELENBP1 in TP53, KRAS, or EGFR‐mutant LUAD patients and TP53, KRAS, or EGFR wild‐type LUAD patients derived from GSE72094 dataset. (C) ROC curves showed the predictive accuracy of TPX2 or SELENBP1 to distinguish TP53‐mutant from TP53 wild‐type LUAD in TCGA and GSE72094 datasets

Previously, we showed the different TP53, KRAS, and EGFR mutations in different sub‐consensuses of LUAD patients (Figure 2D). So, we detected the expression levels of TPX2 and SELENBP1 in TP53, KRAS, or EGFR‐mutant LUAD patients and TP53, KRAS, or EGFR wild‐type LUAD patients derived from TCGA dataset. Compared with TP53 wild‐type LUAD patients, TPX2 was overexpressed in TP53‐mutant LUAD patients (Figure 11A). However, SELENBP1 was downregulated in TP53‐mutant LUAD patients (Figure 11A). Moreover, the upregulations of TPX2 and downregulations of SELENBP1 in TP53‐mutant LUAD patients were confirmed in GSE72094 dataset (Figure 11B). Furthermore, in both TCGA and GSE72094 datasets, TPX2 was downregulated, while, SELENBP1 was upregulated in EGFR‐mutant LUAD patients (Figure 11A,B). However, there was no significant difference in TPX2 and SELENBP1 expression levels between KRAS‐mutant and wild‐type LUAD patients (Figure 11A,B). Next, we tried to determine if TPX2 or SELENBP1 could also distinguish TP53‐mutant from TP53 wild‐type LUAD patients. The ROC analysis showed significant AUC values of TPX2 or SELENBP1 in the prediction of TP53 status in TCGA and GSE72094 datasets (Figure 11C).

## 4 DISCUSSION

Heterogeneity poses great challenges in cancer prognosis and treatment. With the advancements in gene microarray and RNA‐Seq technologies, classification systems based on the transcriptional profiling of cancers are developed. Self‐organizing maps (SOMs) clustering,^52^, ^53^ integrative iCluster clustering,^5^, ^54^, ^55^ network‐based consensus molecular classification (CMS),^56^, ^57^ and nonnegative matrix factorization (NMF) clustering^26^, ^28^, ^31^, ^34^ are all proved to be robust cancer classification systems. However, for the same cohort of tumor patients, distinct clustering methods may result different classification. For example, LUAD patients in TCGA dataset were divided into six clusters by iCluster clustering.^5^ In this study, the same LUAD patients in TCGA dataset were classified into three sub‐consensuses using NMF classification. Both iCluster and NMF method divided the LUAD patients into different groups with distinct molecular characteristics. However, the LUAD patients in different clusters had no significant clinical overall survival, while, sub‐consensus 3 LUAD patients from NMF classification were with lower overall survival than other sub‐consensuses.

Different cohorts of patients and gene expression technologies pose another level of complexity and further influence the classification results.^58^, ^59^ Through same NMF method, LUAD patients from TCGA, GSE30219, GSE50081, GSE68465, and GSE72094 datasets were divided into two‐consensuses with different overall survival. However, in GSE42127 dataset, there was no significantly different overall survival between sub‐consensus 1 and sub‐consensus 2 LUAD patients. Integrating various independent datasets may increase the statistical robustness. So, in this study, we analyzed 1765 LUAD patients from TCGA and five independent GEO datasets, we found that three sub‐consensuses of NMF method was a robust classification method of LUAD. Moreover, TPX2 and SELENBP1 were differentially expressed in the different sub‐consensuses of LUAD patients in TCGA, GSE30219, GSE42127, GSE50081, GSE68465, and GSE72094 datasets. Furthermore, TPX2 and SELENBP1 predicted the inner sub‐consensuses of LUAD with high accuracy.

Except transcriptional profiling, the immune cells infiltration signature or tumor immune microenvironment is another important factor influencing the heterogeneity of LUAD^21^, ^22^ and making it difficult to interpret the results from different LUAD cohorts. On the contrary, classifications of LUAD cell lines may represent the intrinsic heterogeneity of LUAD tumors. In this study, the NMF method was used in the classification of 64 LUAD cell lines. LUAD cell lines in different sub‐consensuses had different responses to EGFR inhibitors. Also, the expression levels of TPX2 and SELENBP1 were different in the different sub‐consensuses of LUAD cell lines.

Previous reports showed that, in NSCLC and in some smoking related LUAD patients, TPX2 was associated with poor prognosis.^60^, ^61^, ^62^ In this study, we further showed that, compared with normal lung tissues, TPX2 was overexpressed in LUAD tissues in TCGA, GSE7670, GSE10072, GSE18842, GSE27262, GSE32863, and GSE30219 datasets. And the overexpression of TPX2 was associated with low relapse‐free survival and low overall survival in TCGA, GSE30219, GSE42127, GSE50081, GSE68465, and GSE72094 datasets. SELENBP1 was reported as a tumor suppressor gene in many types of tumors.^63^, ^64^, ^65^ The prognosis of SELENBP1 in LUAD was unknown. Our data showed that SELENBP1 was downregulated in LUAD tissues and LAUD patients with higher expression levels of SELENBP1 were associated with better relapse‐free survival and overall survival. However, those results were derived from TCGA and GEO datasets, further clinical validations of the prognosis of TPX2 and SELENBP1 were needed. Moreover, the detailed mechanisms of TPX2 and SELENBP1 in LUAD development should be further studied.

## 5 CONCLUSIONS

By integrated analysis of 1765 LUAD patients and 64 LUAD cell lines, we showed that NMF was a robust inner sub‐consensuses classification method of LUAD. Sub‐consensus 3 LUAD patients were with low overall survival and were with high TP53 and KRAS mutations. And sub‐consensus 2 LUAD cell lines were resistant to EGFR inhibitors. TPX2 and SELENBP1 were differentially expressed in different LUAD sub‐consensuses, and predicted the inner sub‐consensuses of LUAD with high accuracy. TPX2 was an unfavorable prognostic biomarker of LUAD which was upregulated in LUAD tissues and associated with the low overall survival of LUAD. SELENBP1 was a favorable prognostic biomarker of LUAD which was downregulated in LUAD tissues and associated with the prolonged overall survival of LUAD.

## CONSENT FOR PUBLICATION

Not applicable.

## CONFLICT OF INTEREST

The authors declare that they have no conflict of interest.

## ETHICS APPROVAL AND CONSENT TO PARTICIPATE

Not applicable.

## Supporting information

Fig S1

Fig S2

Fig S3

## Data Availability

The datasets generated and/or analyzed during the current study are available in TCGA (tcga.xenahubs.net) and GEO (www.ncbi.nlm.nih.gov/geo) repositories.
