# Supplementary figures and images for: Nonnegative matrix factorization‐based bioinformatics analysis reveals that TPX2 and SELENBP1 are two predictors of the inner sub‐consensuses of lung adenocarcinoma
Source: Cancer Med. 2021 Nov 3;10(24):9058–77. doi: 10.1002/cam4.4386 (PMC8683537; doi:10.1002/cam4.4386)

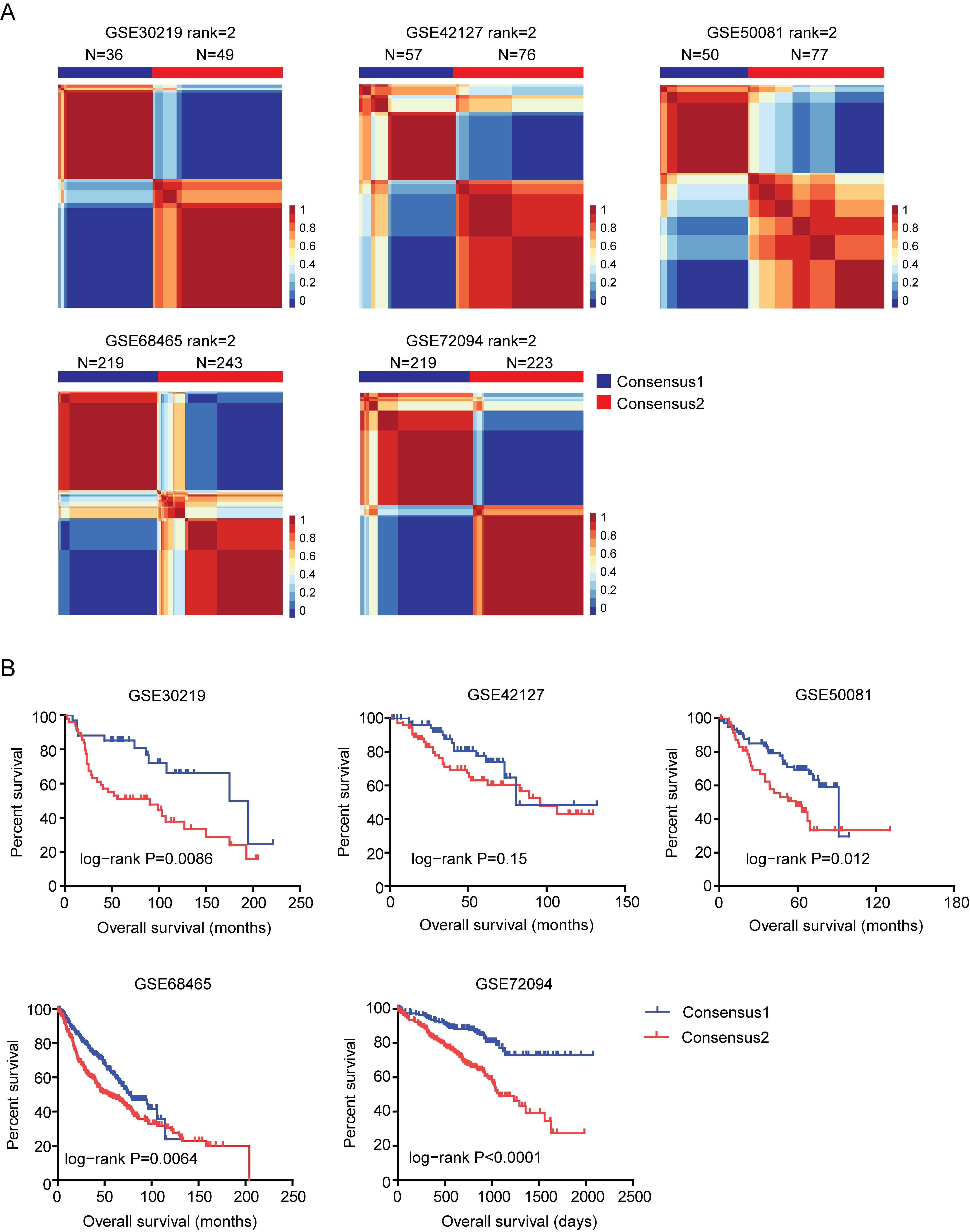

Supplement: Supplementary file 1 — Fig S1 [file CAM4-10-9058-s001.tif]

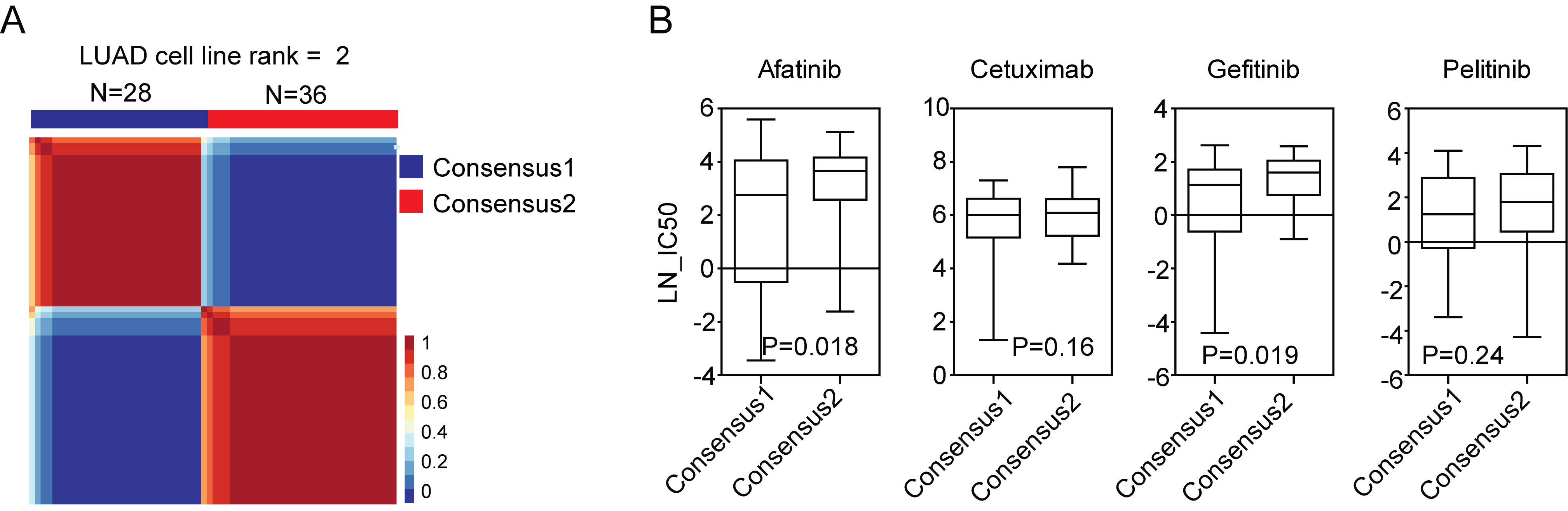

Supplement: Supplementary file 2 — Fig S2 [file CAM4-10-9058-s002.tif]

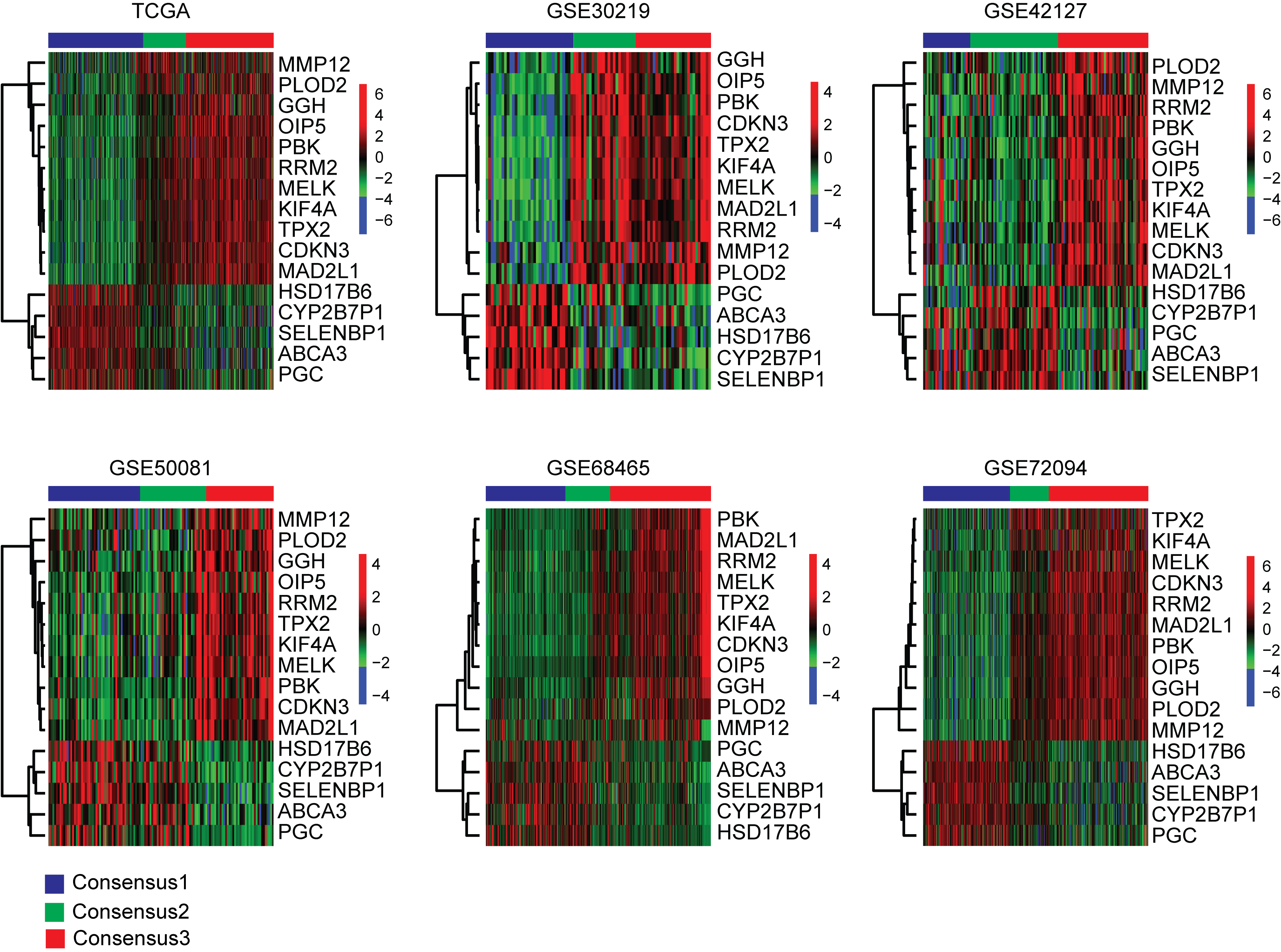

Supplement: Supplementary file 3 — Fig S3 [file CAM4-10-9058-s003.tif]
